# Minimal ovarian stimulation combined with elective single embryo transfer policy: age-specific results of a large, single-centre, Japanese cohort

**DOI:** 10.1186/1477-7827-10-35

**Published:** 2012-04-27

**Authors:** Keiichi Kato, Yuji Takehara, Tomoya Segawa, Satoshi Kawachiya, Takashi Okuno, Tamotsu Kobayashi, Daniel Bodri, Osamu Kato

**Affiliations:** 1 Kato Ladies Clinic, 7-20-3, Nishishinjuku, Shinjuku, Tokyo, 160-0023, Japan; 2 Shinbashi Yume Clinic, Tokyo, Japan

**Keywords:** Minimal stimulation, Natural cycle IVF, Elective single embryo transfer, Assisted reproduction techniques

## Abstract

**Background:**

The two main complications associated with the use of assisted reproduction techniques, ovarian hyperstimulation syndrome and multiple pregnancies, could be eliminated by milder ovarian stimulation protocols and the increased use of a single embryo transfer (SET) policy. A retrospective, cohort study was performed in private infertility centre to evaluate the embryological and clinical results of a large exclusively SET program according to patient age (lower or equal 29, 30–34, 35–39, 40–44 and equal or higher 45 years).

**Materials:**

A total of 7,244 infertile patients have undergone 20,244 cycles with a clomiphene-based minimal stimulation or natural cycle IVF protocol during 2008. Following oocyte retrieval, fertilization and embryo culture a total of 10,401 fresh or frozen single embryo transfer procedures were performed involving cleavage-stage embryos or blastocysts.

**Results:**

Successful oocyte retrieval rate (78.0 %) showed no age-dependent decrease until 45 years. Fertilization (80.3 %) and cleavage (91.1 %) rates were not significantly different between age groups. Blastocyst formation (70.1 % to 22.8 %) and overall live birth rates (35.9 % to 2 %) showed an age-dependent decrease. Frozen-thawed blastocyst transfer cycles gave the highest chance of live birth per embryo transfer (41.3 % to 6.1 %).

**Conclusions:**

High fertilization and cleavage rates were obtained regardless of age whereas blastocyst formation and live birth rates showed an age-dependent decrease. An elective single embryo transfer program based on a minimal ovarian stimulation protocol yields acceptable live birth rates per embryo transfer in infertile patients up until their mid-forties. However in very advanced age patients (equal or higher 45 years old) success rates fall below 1 %.

## Background

In recent years there has been an increasing interest in developing new milder approaches in in-vitro fertilization treatment that might decrease the physical burden and psychological distress for patients, increase patient convenience and contribute to reducing treatment costs [1]. The risks of the two main complications associated with the use of assisted reproduction techniques, ovarian hyperstimulation syndrome and multiple pregnancies, are reduced respectively by milder ovarian stimulation protocols and the increased use of an elective single embryo transfer (SET) policy.

Kato Ladies Clinic (KLC) has pioneered minimal stimulation protocols since 1994 by developing an innovative ovarian stimulation regimen based on clomiphene citrate and triggering final oocyte maturation with a GnRH agonist [2]. More recently, from 2002, an even milder approach was developed and natural cycle IVF was increasingly used for ovulatory women in our centre [3,4]. In this regimen the only pharmaceutical intervention consisted in inducing the final oocyte maturation with a GnRH agonist. Guidelines of the Japanese Society for Reproductive Medicine on the number of transferred embryos (2007) recommended single embryo transfer for good-prognosis patients especially in case of blastocyst transfers. SET has been a routine practice in KLC for more than a decade however a 100 % single embryo transfer policy was implemented in our center for blastocyst transfers from 2006 and for cleavage-stage embryos from 2008.

The purpose of the present study is to report the results of an exclusively single embryo transfer program based on mild ovarian stimulation/natural cycle IVF protocols with over 20,000 started cycles and to analyze outcome according to patient age.

## Methods

### Patients

The study period was between January and December 2008 including all cycles performed in KLC. Institutional Review Board approval was not required for the present study due to its retrospective nature and to the fact that study data was constantly managed in a way which excluded the identification of subjects. Our patient population was composed of infertile patients of advanced mean age (39.4 ± 4.7 years) including many patients with previous failed treatment cycles performed in others centers. A clomiphene based minimal stimulation protocol was used in the majority of our patients whereas from 2002 onwards natural cycle IVF was increasingly offered to normally cycling women.

### Data collection

A dataset was created by summarizing all oocyte retrievals and embryo transfers procedures (including fresh cleavage, fresh blastocyst, vitrified cleavage and vitrified blastocyst transfers) performed during the study period according to five different female age groups (≤29, 30–34, 35–39, 40–44 and ≥45 years). Because it was not possible to link original oocyte retrievals with their respective embryo transfer procedures (when elective cryopreservation occurred with a subsequent frozen-thawed embryo transfer in the next cycle) or to follow-up patients who performed successive stimulation cycles, we could not calculate pregnancy rates per started cycle or cumulative success rates. However an estimate of “per planned oocyte retrieval” success rates was done by dividing all live births (that resulted from different types of embryo transfer procedures) by the number of planned oocyte retrievals in the same age group. This would lead to minimal bias because the only imprecision would result from patients who performed their frozen-thawed embryo transfer outside of the one-year study period or who moved to a different age group during the short time interval between oocyte retrieval and subsequent frozen embryo transfer.

### Minimal stimulation and natural cycle IVF protocol

All patients received detailed information about the proposed treatment option. A written informed consent was obtained from the participants of the study and they have also agreed that their de-identified data could be used for research purposes. A clomiphene citrate (CC) based minimal stimulation protocol was used in the majority of cycles (82 %) whereas unstimulated natural cycle IVF (16.2 %) or letrozole stimulated cycles (1.8 %) were used in a smaller proportion of started cycles. Details of the clomiphene-based minimal stimulation protocol were described previously [2]. Briefly CC (50 to 100 mg/day) was administrated orally with an extended regimen from cycle day 3 until the day before inducing final oocyte maturation. Human menopausal gonadotropin (hMG) or recombinant FSH was added in the form of injections (50 to 150 IU/every other day) or nasal spray in 77.4 % of CC-based stimulation protocols in order to obtain 1 to 4 mature follicles. Monitoring involving ultrasound scan and hormonal profile (E2, LH and progesterone) was usually started on day 8 and continued every other day until triggering day. Ovulation triggering was performed with a GnRH agonist, busereline (Suprecur, 600 μg), administered in a nasal spray form.

In the natural cycle IVF protocol the only pharmaceutical intervention consisted of inducing the final oocyte maturation with a GnRH agonist. Monitoring consisted of an ultrasound scan and hormonal profile (E2, LH and progesterone) and was usually started in the morning of day 10 and/or 12 according to the patient’s cycle length. When the leading follicle reached 18 mm with a concomitant E2 level ≥ 250 pg/ml oocyte retrieval was scheduled. Oocyte retrieval was usually performed 30–34 hours after triggering although in some cases where the start of the LH surge was detected it was anticipated to 20–30 hours after triggering [4].

### Oocyte retrieval, conventional insemination and ICSI procedure

The absence of follicles on ultrasound before starting the oocyte retrieval was assumed to be due to premature ovulation. Oocyte retrieval was performed without anesthesia using an extra-thin 21 G needle (Kitazato, Japan). Follicular flushing was not used during the retrieval. Conventional insemination was performed approximately 3 hours after retrieval and intracytoplasmic sperm injection (ICSI) was performed after a 5 hour interval. The P1/cleavage stage medium supplemented with 10 % SSS (Irvine Scientific, US) was used as a culture medium. ICSI was the preferred insemination method in the presence of moderate/severe male factor infertility, for oocytes that matured in-vitro after oocyte retrieval or in case of previous fertilization failure.

Fertilization assessment was done 16–20 hours after insemination. Normally fertilized zygotes with two pronuclei were cultured individually in a drop of 20 μl of Quinn’s Advantage Protein Plus cleavage medium (SAGE, US) from day 1 to 3. Following this, the embryos were transferred to Quinn’s Advantage Protein Plus blastocyst medium (SAGE, US) from day 4 to 6. All embryos were cultured at 37°C under the gas phase of 5 % O_2_, 5 % CO_2_ and 90 % N_2_ with 100 % humidity in water jacket small multigas incubators (Astec, Japan). The liquid nitrogen was produced by a N_2_ generator system in a 10,000 class clean room environment.

### Embryo culture, frozen-thawed cycles and embryo transfer procedure

During the study period only single embryo transfers were performed in our center and an exclusive SET policy was strictly observed. Patients were counseled accordingly before their treatment started about the risk of multiple pregnancies, the benefits of an elective single embryo transfer and the possibility of embryo cryopreservation. Approximately, half of the embryo transfers (47.1 %) were performed at day 2 or 3 with a fresh cleavage-stage embryo whereas in most of the remaining cases (47.4 %) embryos were cultured to blastocyst-stage and vitrified electively for subsequent use in frozen-thawed blastocyst transfer cycles. Details of the vitrification method using the Cryotop® (Kitazato, Japan) were described previously [5]. Elective frozen-thawed blastocyst transfer was preferred in the presence of tubal factor infertility (tubal obstruction, hydrosalpinx or the history of extrauterine pregnancy) or recurrent implantation failures with cleavage-stage embryos.

Frozen-thawed embryo transfers were performed in spontaneous natural or hormonal replacement cycles. In natural cycles, cleavage-stage embryos and blastocysts were transferred on day 2 and 5 respectively after ovulation was confirmed. In hormonal replacement cycles, transdermal estradiol patches were started from cycle day 2 and dydrogesterone was added from cycle day 11 after which cleavage-stage embryos or blastocysts were transferred 1 or 7 days later, respectively. All embryo transfer procedures were performed under vaginal ultrasound guidance using a specially designed soft catheter (Kitazato, Japan) by placing a single embryo in minimal volume to the mid-uterine cavity [6]. Dydrogesterone (30 mg/day orally) was routinely administered during the early luteal phase both after fresh and frozen-thawed embryo transfer procedures. Moreover intramuscular or intravaginal progesterone was also added until the 9^th^ pregnancy week in cases where the endogenous progesterone production from the placenta was found to be insufficient.

### Outcome measures and pregnancy definitions

Primary outcome measures were clinical pregnancy and live birth rates per embryo transfer. Secondary outcome measures were the rate of successful oocyte retrievals as well as subsequent fertilization, cleavage rates and blastocyst formation rates. Biochemical pregnancy was defined by a serum β-hCG level ≥20.0 mIU/ml approximately 14 days after embryo transfer. An intrauterine gestational sac revealed by ultrasound scan approximately 4 weeks after transfer was considered to be a clinical pregnancy. Miscarriage was defined as a pregnancy loss occurring after detection of a clinical pregnancy up to the 21^st^ week of pregnancy. Multiple pregnancies were defined by the presence of more than one gestational sac or yolk sac or fetal heart beats. Nominal variables were analyzed by the Cochran-Armitage test for trend. P < 0.05 was considered statistically significant.

## Results

Altogether a total of 20,244 cycles were scheduled for oocyte retrieval in 7,244 patients. The mean age of patients undergoing oocyte retrieval was 39.4 ± 4.7 years (range: 22–53 years). The average number of retrieved oocytes per scheduled cycle was 1.54. The rates of premature ovulation detected at oocyte retrieval, successful oocyte retrievals and those with mature oocytes according to different age groups are summarized in 1. No significant differences were observed in the proportion of successful retrievals according to different age groups except a significantly lower rate in ≥45 years old women. Following oocyte retrieval no cases of intra-peritoneal hemorrhage occurred that needed hospitalization. No cases of moderate/severe OHSS were observed following the above-mentioned minimal stimulation/natural cycle IVF protocols which was combined with GnRH agonist trigggering.

**Table 1:** Results of oocyte retrieval according to age

	Age (years)						
	≤29	30-34	35-39	40-44	≥45	**Total**	p^a^
Planned OR, n	448	2751	6595	7600	2850	**20244**	-
Premature ovulations, n (%)	10 (2.23)	65 (2.36)	160 (2.43)	172 (2.26)	60 (2.11)	**476** **(2.35)**	- 0.46
Successful OR, n (%)	372 (83)	2243 (81.5)	5378 (81.5)	5838 (76.8)	1951 (68.5)	**15782** **(78)**	<0.0001
ORs with mature oocyte, n (%)	314 (70.1)	1957 (71.1)	4787 (72.6)	5171 (68)	1698 (59.6)	**13927** **(68.8)**	<0.0001

^a^ Cochran-Armitage test for trend.

The fertilization, cleavage and blastocyst formation rates according to different age groups are summarized in Table 2. No significant differences were observed in fertilization and cleavage rates according to different age groups within the conventional IVF and ICSI groups. However both fertilization (p = 0.001) and cleavage rates (p = 0.003) were slightly higher in the ICSI group compared to conventional IVF. In contrast with the previous two variables the blastocyst formation rate was higher (p < 0.0001) in the conventional IVF group. The blastocyst formation rate showed a statistically highly significant age-dependent decline (p < 0.0001).

**Table 2:** Embryological results according to age

	Age (years)						
	≤29	30-34	35-39	40-44	≥45	**Total**	p^a^
Fertilization rate							
IVF, %	76.6	73.6	76.1	79.9	77.7	**77**	0.07
ICSI, %	85.4	83.7	83.1	83.1	83	**83.2**	0.75
	Cleavage rate							
IVF, %	92.4	89.2	87.5	88.5	87.7	**88.3**	0.7	
ICSI, %	93.9	95	94.7	92.7	90.2	**93.4**	0.24	
IVF, %	70.5	67	64.4	49.7	25.9	**57.3**	<0.0001	
ICSI, %	69.8	59.5	56	40.1	21.7	**46.6**	<0.0001	

^a^ Cochran-Armitage test for trend.

**Figure 1:**
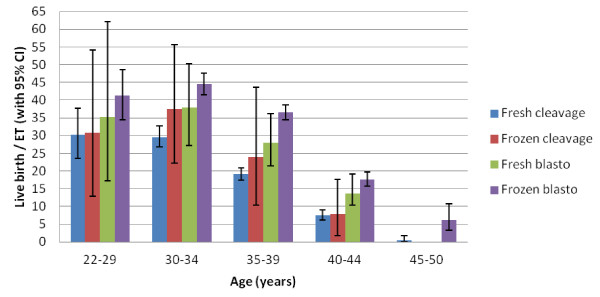
Age-specific live birth rates per embryo transfer in different embryo transfer groups (with 95 % CI).

Out of a total 10,401 performed embryo transfer procedures, 4,902 (47.1 %) were fresh cleavage-stage embryo transfers, 149 (1.4 %) fresh blastocyst transfers, 416 (4 %) frozen-thawed cleavage-stage embryo transfers and 4,934 (47.4 %) frozen-thawed blastocyst transfers. The mean age of patients undergoing embryo transfer was 38.1 ± 4.1 years (range: 22–50 years). Average survival rates after thawing were 98 % for vitrified cleavage-stage embryos and 95 %, and for vitrified blastocysts. Clinical pregnancy and live birth rates per embryo transfer according to five age groups are summarized in Table 3. An age-dependent decrease in live birth rates was observed after 35 years in all embryo transfer groups which was statistically highly significant in all except the fresh blastocyst group with the smallest sample size. The oldest patient achieving a live birth in this series was 48 years old. Compared to other groups the frozen-thawed blastocyst transfer resulted in the highest live birth rates in all age groups (Figure 1). An estimation of total live birth rates per planned oocyte retrieval showed that success rates decreased gradually from 30 to 0.46 % in ≤29 years up to ≥ 45 years old patients, respectively (Table 4.)

**Table 3:** Pregnancy and live birth rates per embryo transfer according to age

							
	≤29	30-34	35-39	40-44	≥45	**Total**	p^a^
Fresh embryo transfer, n	159	931	1972	1428	412	**4902**	-
Clinical pregnancies, n (%)	53 (33.3)	324 (34.8)	507 (25.7)	179 (12.5)	4 (1)	**1067** **(21.8)**	<0.0001
Live births, n (%)	48 (30.2)	275 (29.5)	376 (19.1)	106 (7.4)	2 (0.5)	**807** **(16.5)**	<0.0001
Fresh blastocyst transfer, n	17	29	25	73	5	**149**	-
Clinical pregnancies, n (%)	6 (35.3)	15 (51.7)	9 (36)	20 (27.4)	2 (40)	**52** **(34.9)**	0.29
Live births, n (%)	6 (35.3)	11 (37.9)	7 (28)	10 (13.7)	1 (20)	**35** **(23.5)**	0.036
Vitrified embryo transfer, n	13	64	125	153	61	**416**	-
Clinical pregnancies, n (%)	5 (38.5)	33 (51.6)	38 (30.4)	18 (11.8)	2 (3.3)	**95** **(22.8)**	<0.0001
Live births, n (%)	4 (30.8)	24 (37.5)	30 (24)	12 (7.8)	0 (0)	**70** **(16.8)**	<0.0001
Vitrified blastocyst transfer, n	184	1024	2072	1489	165	**4934**	-
Clinical pregnancies, n (%)	93 (50.5)	553 (54)	998 (48.2)	471 (31.6)	33 (20)	**2149** **(43.6)**	<0.0001
Live births, n (%)	76 (41.3)	456 (44.5)	757 (36.5)	263 (17.7)	10 (6.1)	**1562** **(31.7)**	<0.0001

^a^ Cochran-Armitage test for trend.

**Table 4:** Estimated total live birth rates per planned oocyte retrieval according to age

							
	≤29	30-34	35-39	40-44	≥45	**Total**	p^a^
Planned OR, n	448	2751	6595	7600	2850	**20244**	-
Total embryo transfers n, (%)	373 (83.3)	2048 (74.4)	4194 (63.6)	3143 (41.4)	643 (22.6)	**10401** **(51.4)**	<0.0001
Total live births n, (%)	134 (30)	766 (27.8)	1170 (17.7)	391 (5.1)	13 (0.46)	**2474** **(12.2)**	<0.0001

^a^ Cochran-Armitage test for trend.

Overall multiple pregnancy rates were 0.9 % consisting of monozygotic twins, which is almost comparable to the overall twinning rate of Japanese population. Extrauterine pregnancy rates were significantly lower after single blastocyst transfer 0.36 % (9/2,523) compared to cleavage-stage embryos 1.41 % (19/1,351), p < 0.0001.

## Discussion

The present retrospective cohort study demonstrates that an elective single embryo transfer program based on the use of minimal stimulation/natural cycle IVF protocols yields good embryological results (fertilization, cleavage, blastocyst formation rates) and leads to acceptable live birth rates in infertile patients of advanced age up until their mid-forties.

Successful oocyte retrieval rates - with the obtention of at least one mature oocyte - were not affected until 45 years of age and then drop by only approximately 10 %. As the majority of cycles were clomiphene-based this might be related to the fact that premature LH surges were efficiently blocked by the extended CC regimen [7]. In comparison in a recent series the rate of successful oocyte retrievals was lower in nIVF cycles compared to the observed retrieval rate in our study (66 % versus 78 %) [4]. This suggests that natural cycle IVF is especially prone to high cancellation rates due to premature LH surges and the loss of a single follicle following premature ovulation detected at the retrieval.

Fertilization and cleavage rates were high regardless of patient age. Overall fertilization rates were slightly (5-6 %) higher with ICSI which was described previously [8]. Therefore in the context of minimal ovarian stimulation protocols where only a single or a few mature oocytes are available the routine use of ICSI might be beneficial. Conversely blastocyst formation rates were significantly lower after ICSI which might be related to a higher proportion of male factor infertility among couples requiring ICSI [9]. Both blastocyst formation and subsequent live birth rates showed a highly significant age dependent decrease which is related to an inevitable decline in oocyte quality. Nonetheless the good overall embryological results might be related to the beneficial effect of milder stimulation protocols. It was previously demonstrated that the proportion of euploid embryos are significantly higher following milder compared to conventional high-dose ovarian stimulation [10]. Furthermore mild approaches might also improve endometrial receptivity and luteal function which are altered following conventional ovarian stimulation. It is interesting to note that in our series, higher clinical pregnancy and live birth rates were obtained in frozen-thawed embryo cycles compared to fresh cycles both with cleavage-stage embryos and blastocysts (Figure 1). This might be related to the potential anti-oestrogenic effect of CC on the endometrial level in fresh cycles which, in contrast, is abolished in natural cycle/hormonal replacement frozen-thawed embryo transfer cycles.

Not surprisingly and in line with previous studies [11,12] an age-related decline in success rates was also demonstrated in our series but in the setting of minimal ovarian stimulation. This is an important issue because our patient population is particularly biased for advanced aged women. In fact more than half (52 %) of the cycles involved ≥40 year-old infertile patients and a non-negligible proportion (14 %) represented very advanced age (≥45 years-old) women. A rough estimation of the overall efficiency of minimal ovarian stimulation is possible by comparing age-specific live birth rates per embryo transfer from our series with large US registry cohorts such as the Society for Assisted Reproductive Technology (SART, http://www.sart.org) data from 2008. Although success rates of our fresh cleavage-stage SET group are generally lower than SART data, the frozen-thawed SBT group yields very close or in some older age groups probably even higher results than overall results of the US cohort (with transfer of 2.2-3.3 embryos). A less biased comparison made with another European SET series [13]- including 36–39 year old patients where conventional ovarian stimulation was used - suggests that after one transfer cycle live birth rates per embryo transfer are comparable (27.9 % versus 26 %) with the two approaches. The very advanced age (≤45 years) patient group represents a particular challenge especially in Japan where oocyte donation treatment is not practiced. In our series for this age group live birth rates per started cycle have fallen below 1 % which is qualified as a “futile treatment” by recent ASRM guidelines [14]. Nonetheless those few patients who are still able to perform a frozen-thawed blastocyst embryo transfer could achieve a somewhat more encouraging 6 % success rate. In fact most (10/13) live births in very advanced age patients in our series were obtained with this approach. In contrast it seems that less complex milder approaches such as natural cycle IVF might reach their limit in very advanced aged women.

A peculiarity of both protocols (clomiphene based and natural cycle) used in this series is that the induction of final oocyte maturation was performed with a GnRH agonist instead of hCG. To our knowledge no other group has used this approach routinely in minimal stimulation or natural cycle IVF protocols. In other clinical situations such as high-responder IVF patients or oocyte donors GnRH agonist triggering was shown to practically eliminate any risk of moderate/severe OHSS [15]. It was also suggested that avoiding hCG might have additional beneficial effects on the maturation rate of obtained oocytes [16] or luteal function [2].

An important advantage of an exclusively single embryo transfer program is the dramatic reduction in multiple gestation rates. In the previously mentioned 2008 SART cohort involving non-donor IVF cycles multiple gestation rates varied, according to age, between 15.4-33.3 % for twins and 0.6-1.9 % for triplets and occurred at all groups without exception. In the setting of a single embryo transfer program only monozygotic twinning (MZT) occurs. In our series MZT rate was low (0.9 %) compared to other reports and might be associated to blastocyst culture [17].

In our series a reduced rate of extrauterine pregnancy was observed following blastocyst transfer in comparison with cleavage-stage embryos. It was suggested that a higher embryonic implantation potential and a lower number of transferred embryos are factors that diminish the risk of an ectopic pregnancy after assisted reproductive techniques[18].

Limitations of the present study are related to potential heterogeneity in patient characteristics, stimulation protocols used or the presence of four different embryo transfer strategies which is inevitable when evaluating such a large number of cycles. Exact live birth rates are presented “per embryo transfer” whereas only an estimation could be performed to show “per cycle” success rates which take into account the proportion of cancelled cycles that finally did not reach embryo transfer. Moreover cumulative live were not presented in this study because it was not possible to link successive patient cycles performed over a relatively short one-year study period. On the other hand this large one year cohort from a single-centre gives a useful insight into the overall efficiency of minimal stimulation protocols combined with a strict SET policy.

## Conclusions

The present study shows that an elective single embryo transfer program based on minimal stimulation ovarian protocols can yield acceptable live birth rates per embryo transfer in infertile patients up until their mid-forties. In very advanced age patients (≥45 years old) however per cycle success rates fall below 1 %. The combination of mild stimulation protocols, a highly efficient embryo cryopreservation technique and a single embryo transfer policy could provide the base for establishing a new concept of innovative, patient-friendly and low-risk in-vitro-fertilization treatment. Further prospectively designed studies stretching over several years are needed to evaluate age-specific cumulative live birth rates after a series of successive treatment cycles using minimal ovarian stimulation.

## Competing interests

The authors declare that they have no competing interests.

## Authors’ contributions

KK contributed to study design, data acquisition, analysis and interpretation, critical review of the manuscript, YT TS SK TO TK and OK contributed to study design and critical review of the manuscript, DB contributed to study design, analyzed the data and drafted the manuscript. All authors read and approved the final version of the manuscript.

## References

[B1] Devroey P Aboulghar M Garcia-Velasco J Griesinger G Humaidan P Kolibianakis E Ledger W Tomas C Fauser BC Improving the patient's experience of IVF/ICSI: a proposal for an ovarian stimulation protocol with GnRH antagonist co-treatment Hum Reprod 2009 24 764 774 19153090 10.1093/humrep/den468

[B2] Teramoto S Kato O Minimal ovarian stimulation with clomiphene citrate: a large-scale retrospective study Reprod Biomed Online 2007 15 134 148 10.1016/S1472-6483(10)60701-8 17697488

[B3] Matsuura T Takehara Y Kaijima H Teramoto S Kato O Natural IVF cycles may be desirable for women with repeated failures by stimulated IVF cycles J Assist Reprod Genet 2008 25 163 167 10.1007/s10815-008-9204-7 18297389 PMC2582079

[B4] Kawachiya S Matsumoto T Bodri D Kato K Takehara Y Kato O Short-term, low-dose, non-steroidal anti-inflammatory drug application diminishes premature ovulation in natural-cycle IVF Reprod Biomed Online 24 308 313 22285246 10.1016/j.rbmo.2011.12.002

[B5] Kuwayama M Vajta G Kato O Leibo SP Highly efficient vitrification method for cryopreservation of human oocytes Reprod Biomed Online 2005 11 300 308 10.1016/S1472-6483(10)60837-1 16176668

[B6] Bodri D Colodron M Garcia D Obradors A Vernaeve V Coll O Transvaginal versus transabdominal ultrasound guidance for embryo transfer in donor oocyte recipients: a randomized clinical trial Fertil Steril 95 2263 2268 2268 e2261 21459374 10.1016/j.fertnstert.2011.03.028

[B7] Kawachiya S Segawa T Kato K Takehara Y Teramoto S Kato O The effectiveness of clomiphene citrate in supressing the LH surge in the minimal stimulation protocol Fertil Steril 2006 86 412 751

[B8] Orief Y Dafopoulos K Al-Hassani S Should ICSI be used in non-male factor infertility? Reprod Biomed Online 2004 9 348 356 10.1016/S1472-6483(10)62152-9 15353089

[B9] Tesarik J Paternal effects on cell division in the human preimplantation embryo Reprod Biomed Online 2005 10 370 375 10.1016/S1472-6483(10)61798-1 15820045

[B10] Baart EB Martini E Eijkemans MJ Van Opstal D Beckers NG Verhoeff A Macklon NS Fauser BC Milder ovarian stimulation for in-vitro fertilization reduces aneuploidy in the human preimplantation embryo: a randomized controlled trial Hum Reprod 2007 22 980 988 10.1093/humrep/del484 17204525

[B11] Baird DT Collins J Egozcue J Evers LH Gianaroli L Leridon H Sunde A Templeton A Van Steirteghem A Cohen J Fertility and ageing Hum Reprod Update 2005 11 261 276 15831503 10.1093/humupd/dmi006

[B12] van Loendersloot LL van Wely M Limpens J Bossuyt PM Repping S van der Veen F Predictive factors in in vitro fertilization (IVF): a systematic review and meta-analysis Hum Reprod Update 16 577 589 20581128 10.1093/humupd/dmq015

[B13] Veleva Z Vilska S Hyden-Granskog C Tiitinen A Tapanainen JS Martikainen H Elective single embryo transfer in women aged 36–39 years Hum Reprod 2006 21 2098 2102 10.1093/humrep/del137 16740524

[B14] Fertility treatment when the prognosis is very poor or futile Fertil Steril 2009 92 1194 1197 19726040 10.1016/j.fertnstert.2009.07.979

[B15] Bodri D Guillen JJ Galindo A Mataro D Pujol A Coll O Triggering with human chorionic gonadotropin or a gonadotropin-releasing hormone agonist in gonadotropin-releasing hormone antagonist-treated oocyte donor cycles: findings of a large retrospective cohort study Fertil Steril 2009 91 365 371 10.1016/j.fertnstert.2007.11.049 18367175

[B16] Humaidan P Papanikolaou EG Tarlatzis BC GnRHa to trigger final oocyte maturation: a time to reconsider Hum Reprod 2009 24 2389 2394 10.1093/humrep/dep246 19608565

[B17] Kawachiya S Bodri D Shimada N Kato K Takehara Y Kato O Blastocyst culture is associated with an elevated incidence of monozygotic twinning after single embryo transfer Fertil Steril 95 2140 2142 21215395 10.1016/j.fertnstert.2010.12.018

[B18] Chang HJ Suh CS Ectopic pregnancy after assisted reproductive technology: what are the risk factors? Curr Opin Obstet Gynecol 22 202 207 20216415 10.1097/GCO.0b013e32833848fd

